# Automated and real-time segmentation of suspicious breast masses using convolutional neural network

**DOI:** 10.1371/journal.pone.0195816

**Published:** 2018-05-16

**Authors:** Viksit Kumar, Jeremy M. Webb, Adriana Gregory, Max Denis, Duane D. Meixner, Mahdi Bayat, Dana H. Whaley, Mostafa Fatemi, Azra Alizad

**Affiliations:** 1 Department of Physiology and Biomedical Engineering, Mayo Clinic College of Medicine and Science, Rochester, Minnesota, United States of America; 2 Department of Radiology, Mayo Clinic College of Medicine and Science, Rochester, Minnesota, United States of America; University of Pennsylvania Perelman School of Medicine, UNITED STATES

## Abstract

In this work, a computer-aided tool for detection was developed to segment breast masses from clinical ultrasound (US) scans. The underlying Multi U-net algorithm is based on convolutional neural networks. Under the Mayo Clinic Institutional Review Board protocol, a prospective study of the automatic segmentation of suspicious breast masses was performed. The cohort consisted of 258 female patients who were clinically identified with suspicious breast masses and underwent clinical US scan and breast biopsy. The computer-aided detection tool effectively segmented the breast masses, achieving a mean Dice coefficient of 0.82, a true positive fraction (TPF) of 0.84, and a false positive fraction (FPF) of 0.01. By avoiding positioning of an initial seed, the algorithm is able to segment images in real time (13–55 ms per image), and can have potential clinical applications. The algorithm is at par with a conventional seeded algorithm, which had a mean Dice coefficient of 0.84 and performs significantly better (P< 0.0001) than the original U-net algorithm.

## Introduction

Breast cancer is the most common cancer among American women after skin cancer, and is a leading cause of death with an estimate of 40,450 cases in 2016 [1, 2]. Additionally, more than half of the cases of breast cancer occur in the developing world, with a mortality rate inversely related to the country’s wealth [2]. Various imaging modalities are used for screening breast tissue with the goal of early detection (e.g., mammography, US, and magnetic resonance imaging). Annual breast cancer mammography screening is recommended by the American Cancer Society for women between the ages of 45 and 54, with biennial screening after the age of 54 [3]. Mammography is discouraged in women younger than 45 due to the risk radiation poses and likelihood of outweighed benefits. Furthermore, younger, premenopausal women have denser breasts compared to older, postmenopausal women, which makes interpretation of mammograms more difficult [4]. Young patients with high breast density and family history of cancer often undergo magnetic resonance imaging or US examination for cancer screening, thus, US plays a major role for patients who cannot undergo mammography examination [5]. In addition, US is commonly used as a secondary screening modality to further inspect suspicious breast masses identified by mammography. US imaging is a relatively inexpensive, noninvasive, and widely available medical imaging technique used for breast cancer screening, with growing use in developing countries [6]. Ultrasonographers use the morphological and textural features to identify suspicious masses. These visual cues can be shape, margin, echo pattern, posterior features, presence of calcifications, or architectural distortion [7]. The suspicious mass is then scored based on the Breast Imaging Reporting and Data System (BI-RADS) scale. The BI-RADS scale is a purely visual system developed to quantify cancer suspicion in breast masses and is the basis for recommending core needle biopsy or continuous monitoring if the mass is suspected to be of low suspicion. With the aid of mammography, localization of breast masses with US is done relatively effortlessly; however, in the absence of a mammogram, many challenges arise when finding breast masses only with US, as US requires a meticulous scanning of the entire breast. A computer-aided detection system could reduce the time sonographers spend finding breast masses, thus making the localization and segmentation process more efficient.

Algorithms to segment breast masses on ultrasound imaging in two and three dimensions have been proposed. The majority of the algorithms use a seeded boundary, which is a rough estimate of the mass boundary drawn on a single B-mode frame or an initial point seed to initiate the segmentation algorithm. Some examples include, a leak plugging algorithm to find diffused and partially diffused boundaries based on a pre-specified seed [8, 9], region-growing algorithms that grow regions based on an initial seed and eventually converge to the segmented boundaries [9–13], active contour model and its variations [14–16], a level set algorithm which uses the principle of active contour energy minimization [17–19], a two-stage active contour method based on an initial point seed [20], an automated particle swarm optimization clustering algorithm which does not require an initial seed but is computationally costly and not suitable for live imaging implementation [21], and a segmentation algorithm based on the cellular automata principle which requires an initial seed [22]. Marking a seed is a trivial task when reviewing cases retrospectively, but is a major impediment for segmentation during live imaging. Correct segmentation of breast masses is very important, as determining malignancy of a mass is critically dependent on the mass morphological features (e.g., shape, smoothness of boundary). Therefore, any automated approach to classify breast masses should first be able to accurately identify the mass boundary.

Deep learning takes advantage of improvements in graphics processing unit’s computing power to develop larger and more complex neural networks capable of performing vision-based tasks comparable to humans and, in some cases, exceeding human performance [23]. Deep learning algorithms have been previously used for classification of benign and malignant breast masses [24, 25]. In this paper, we propose a Multi U-net algorithm to segment suspicious breast masses on US imaging. The proposed algorithm builds up on existing deep learning based segmentation algorithm [26]. The segmentation algorithm is introduced first, followed by implementation on 258 patients and comparison with conventional seed based algorithm and original U-net algorithm.

## Materials and methods

### Patients

Clinical US Images were taken using 2 different commercial clinical US machines: LOGIQ E9 (General Electric; Boston, USA) and IU22 (Philips; Amsterdam, Netherlands). No specific probe, center frequency, or gain settings were specified for image acquisition. Written consent was obtained from all patients along with proper institutional review board approval from Mayo Clinic, while being HIPPA complaint. Patients older than age 19 undergoing biopsy after US imaging for breast cancer were included in the prospective study. Patients with breast implants, abnormalities, and who previously underwent any breast surgical procedures were excluded from the study. A total of 258 patients participated, resulting in 433 US clinical images from multiple orientations. US images with calipers or region of interest (e.g., boxes) were excluded. One hundred twenty-four (124) masses were malignant and one hundred thirty-four masses (134) were benign, as confirmed by biopsy. Table 1 shows the distribution of BI-RADS among the patient population. Most of the cases were BI-RADS 4, which are suspicious cases as they do not present clear features of benignity or malignancy.

**Table 1:** BI-RADS distribution of patients in training/validation and test sets.

BI-RADS	No. of Patients in Training and Validation Set	No. of Patients in Testing Set
2	3	1
3	2	10
4	155	35
5	41	15
6	6	0

Table 2 shows the number of patients for all seven types of malignant pathologies. Table 3 displays the number of patients for twelve different benign pathologies. The other benign pathologies include 1 case each of: apocrine metaplasia, complex sclerosing lesion, diabetic mastopathy, fibroadipose tissue, fibroconnective adipose tissue, fibrin deposition, fibroadenomatoid, fibromyxoid spindle cell lesion, hematoma, histiocytes, intramammary lymph node, mastitis, and papillary proliferation. The data was divided into three groups: training, validation, and testing. Images reserved for testing was not used in the training and validation set. The training set consisted of 337 images, the validation set consisted of 35 images, and the test set consisted of 61 images. The sets were divided such that individual patients appear in only one set. Images were manually segmented by a trained sonographer with thirty one years of experience and were used as gold standard. Identifying breast mass boundaries is a subjective process; therefore, having an experienced professional is of critical importance.

**Table 2:** Number of patients with malignant pathologies.

Malignant Pathologies	Sample size, n
Ductal carcinoma in situ	5
Invasive ductal carcinoma (IDC)	76
Invasive lobular carcinoma (ILC)	14
Invasive mammary carcinoma (IMC)	28
Lymphoma	1
Serous carcinoma	1
Metaplastic carcinoma	1

**Table 3:** Number of patients with benign pathologies.

Benign Pathologies	Sample size, n
Cellular fibroepithelial lesion	2
Clustered apocrine cysts	7
Cyst	3
Fat necrosis	9
Fibroadenoma	52
Fibrocystic changes	12
Fibrosis	9
Hyperplasia	2
Others	19
Papilloma	16
Parenchyma	6
Pseudoangiomatous stromal hyperplasia	3
Sclerosis	2

### Preprocessing

The clinical US images were down sampled to 208 by 208 pixels with zero padding to preserve the image aspect ratio. The US images were taken with different imaging voltages, gain settings, and different transducers, thus resulting in variation in B-mode intensity values. To standardize the images, standard scores for each image were calculated by subtracting the mean value of the image from each pixel followed by division with the standard deviation of the image.

### Algorithm

**Fig 1:**
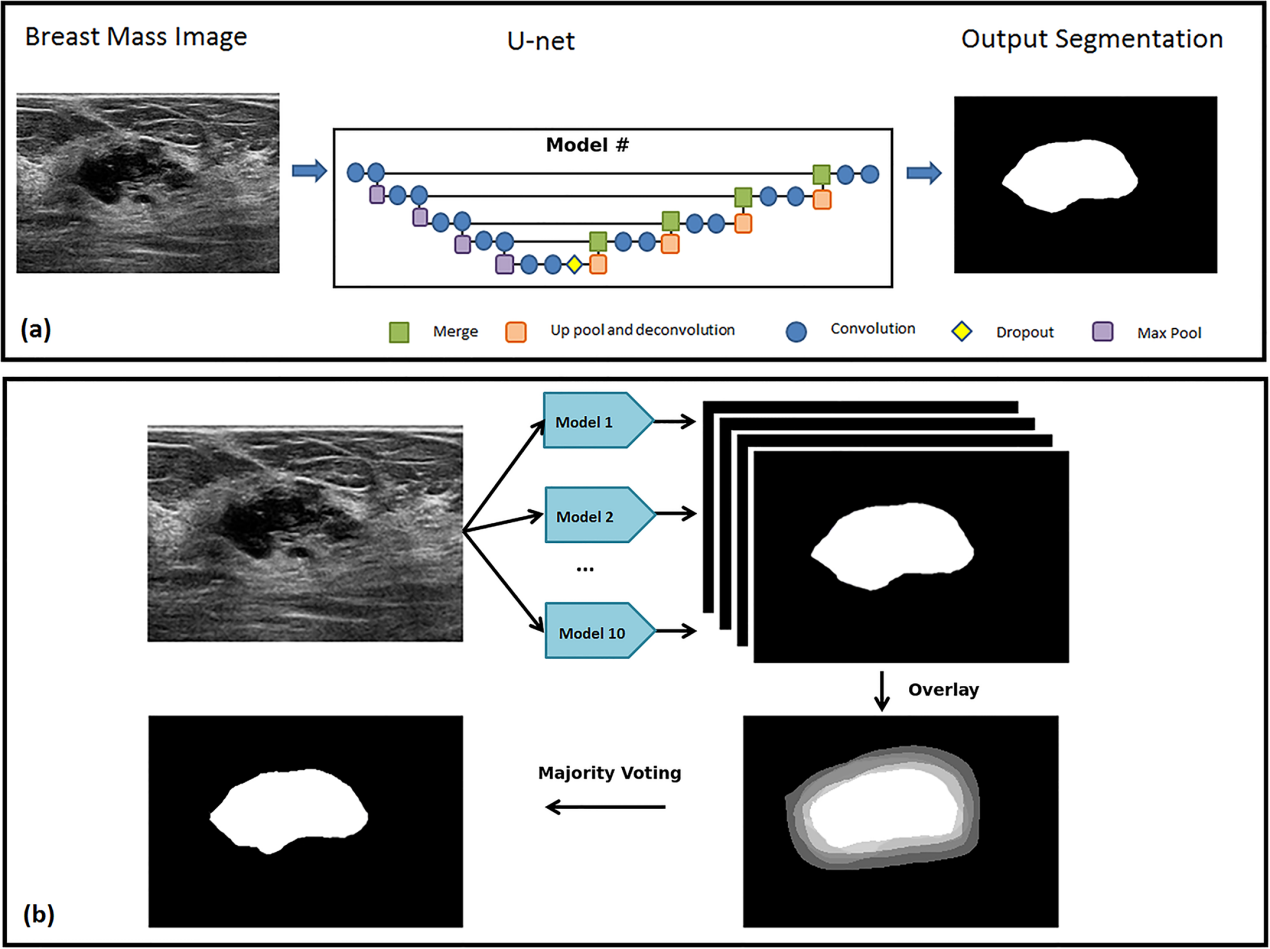
Architecture of (a) Original U-net algorithm, (b) Multi U-net algorithm used to segment suspicious breast masses. Multi U-net consists of 10 models architecturally similar to original U-net followed by majority voting to have a single binary segmentation mask.

Fig 1(a) illustrates the original U-net algorithm [26]. Fig 1(b) summarizes the use of multiple U-net algorithms to create a single segmentation mask by using majority voting on the inputs of multiple U-net. The U-net algorithm consists of the feature collecting encoding branch on the left side and the rebuilding decoding branch on the right side. The encoding branch consists of five layers, and each layer has two convolutional layers with a nonlinear activation function using leaky rectified linear units [27]. The essential parameters of the Multi U-net algorithm are summarized in Table 4 along with the corresponding parameters for original U-net. The Max-pooling layer reduces the dimensionality of the resulting output, enabling further collection of features. At the deepest layer (layer 5), a dropout layer is used to randomly drop out filters to avoid overfitting. After collecting the required features, the decoding branch of Multi U-net performs nonlinear up sampling of the feature maps before merging with a skip connection from the encoding branch. The final output is obtained by passing the result of last decoder through a sigmoid classifier, which independently assigns a probability to each pixel. The input to the algorithm is a preprocessed, down sampled B-mode image and the output is a probability map with predicted suspicious mass and predicted normal breast tissue. The algorithm was developed using Python (version 2.7.11, Python Software Foundation) and open-source Keras Python library (version 1.1.0). The algorithm was executed on a Tesla K40C (Nvidia; Santa Clara, USA) graphic processing unit. A ten-fold cross-validated Multi U-net model is used in which the data is split into 9:1 parts, with 9 parts used for training and 1 part used for validation. Thus, ten unique U-nets are trained and all individual U-nets are randomly initialized. S1 Fig in appendix describes the cross validation technique in more detail.

**Table 4:** Parameters for deep learning algorithms.

Parameter	Multi U-net	Orignal U-net
Convolution size, stride, padding	3x3,1x1, zero padding	3x3, 1x1, no padding
Maximum pooling size, stride, padding	2x2, 2x2, zero padding	2x2, 2x2, no padding
Dropout	0.6	0.5
Up sampling size	2x2	2x2
Optimizer, learning rate	RMSprop [28], 5x10^-6^	SGD, momentum = 0.99
Loss function	Negative Dice coefficient	Categorical crossentropy
Number of filters for convolutional layer	2^(5 + layer number)^	2^(5 + layer number)^
Layer initialization	LeCun uniform [29]	Xavier normal
Cropping size per edge (layer#)	None	88(1), 40(2), 16(3), 4(4)
Image size	208x208	572x572

### Data augmentation

One of the major concerns when using deep learning is overfitting; this is particularly true for convolutional neural networks [30]. The features in US images are dependent upon the interaction of US waves and the tissue, like acoustic shadowing and size of the suspicious mass. While augmenting data these US features must be preserved. Thus, horizontal flipping and equal axis zooming are the only data augmentation techniques used.

### Post-processing

Post-processing was used to improve performance of the network. Equally weighted binary pixels from the ten-fold cross-validated Multi U-net models were averaged and a threshold was used to implement majority voting. A majority voting threshold of 0.5 was used, as justified later in the discussion section. Majority voting removes uncertainty of finding the minima associated with random initialization of the individual U-nets.

### Segmentation evaluation

The proposed algorithm was evaluated using the Dice coefficient (similarity index), true positive fraction (TPF), and false positive fraction (FPF). All three parameters range between 0 and 1; values closer to 1 are better for the Dice coefficient and TPF, and values closer to 0 are better for FPF. Box plot distributions showing the performance of the above mentioned three parameters against different pathologies and BI-RADS were also examined. The dominant pathologies of benign and malignant cases were additionally analyzed separately (i.e., fibroadenoma and invasive ductal carcinoma (IDC), respectively).

### Comparison with conventional seeded algorithm and original U-net algorithm

To compare the performance of Multi U-net algorithm with conventional seeded algorithm, a distance regularized level set segmentation (DRLS) algorithm [19] was implemented. Similar to Multi U-net algorithm the clinical images were down sampled to 208 by 208 pixels. The initial seed was created by eroding the true mask by either 4 or 8 pixels depending on the size of the lesion. Lesions having less than 500 pixels in total were eroded by 4 and lesions having greater than 500 pixels were eroded by 8. To best estimate the parameters of the algorithm an initial random search was performed followed by a finer grid search in the neighborhood of the best performing parameters. The optimal parameters obtained from the search were lambda = 0.5, alpha = -0.75, epsilon = 0.5; as defined by Chunming Li et al. [19]. To highlight the difference between the original U-net [26] and the Multi U-net algorithm the images are also segmented using the original U-net with the parameters as mentioned in Table 4.

## Results

**Fig 2:**
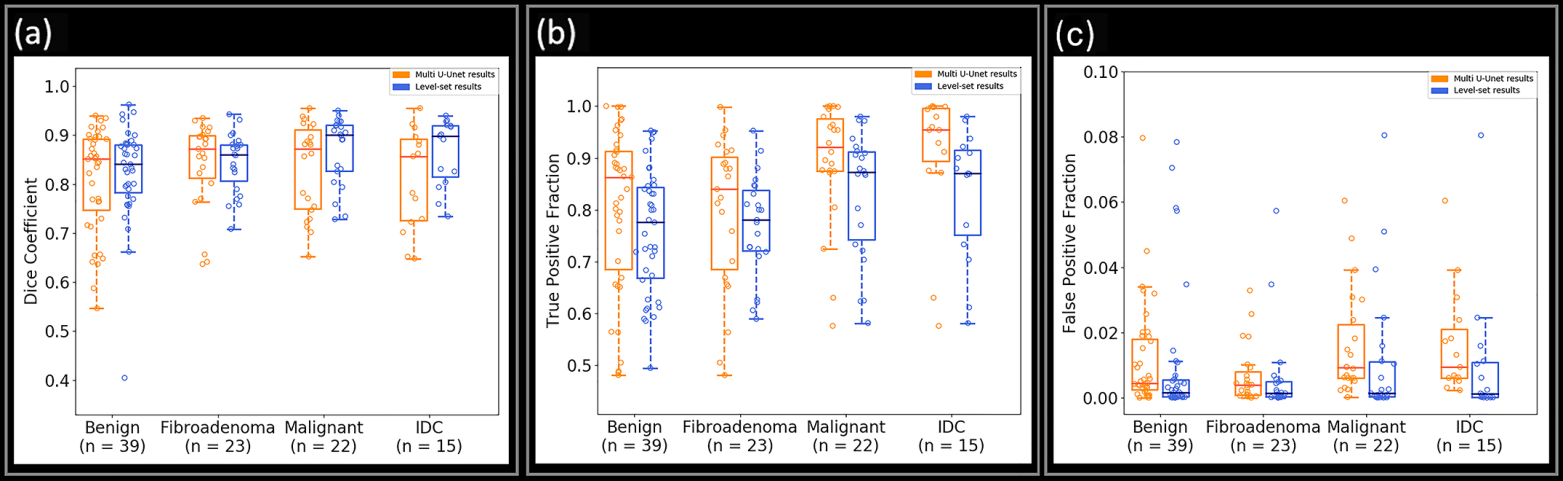
Boxplot showing the performance of Multi U-net and DRLS algorithm for (a) Dice Coefficient, (b) TPF, and (c) FPF for benign, fibroadenoma, malignant, and invasive ductal carcinoma. TPF indicates true positive fraction; FPF indicates false positive fraction.

**Fig 3:**
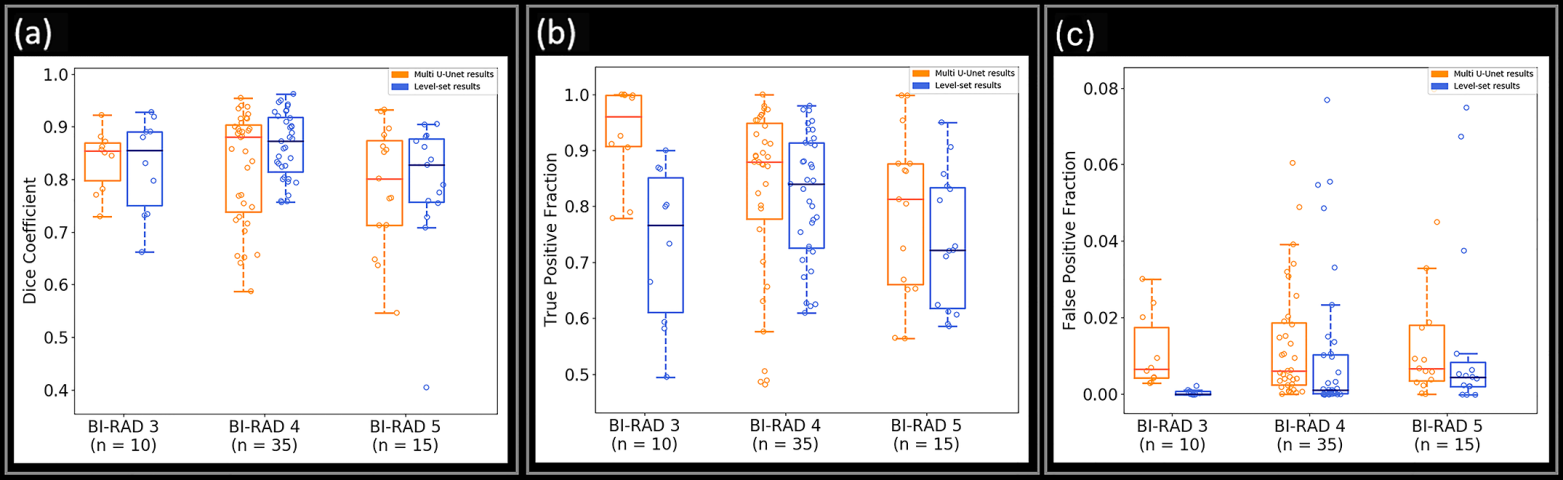
Boxplot showing the performance of Multi U-net and DRLS algorithm for (a) Dice coefficient, (b) TPF, and (c) FPF for BI-RADS 3, 4 and 5. BI-RADS indicate Breast Imaging Reporting and Data System. TPF indicates true positive fraction; FPF indicates false positive fraction.

**Fig 4:**
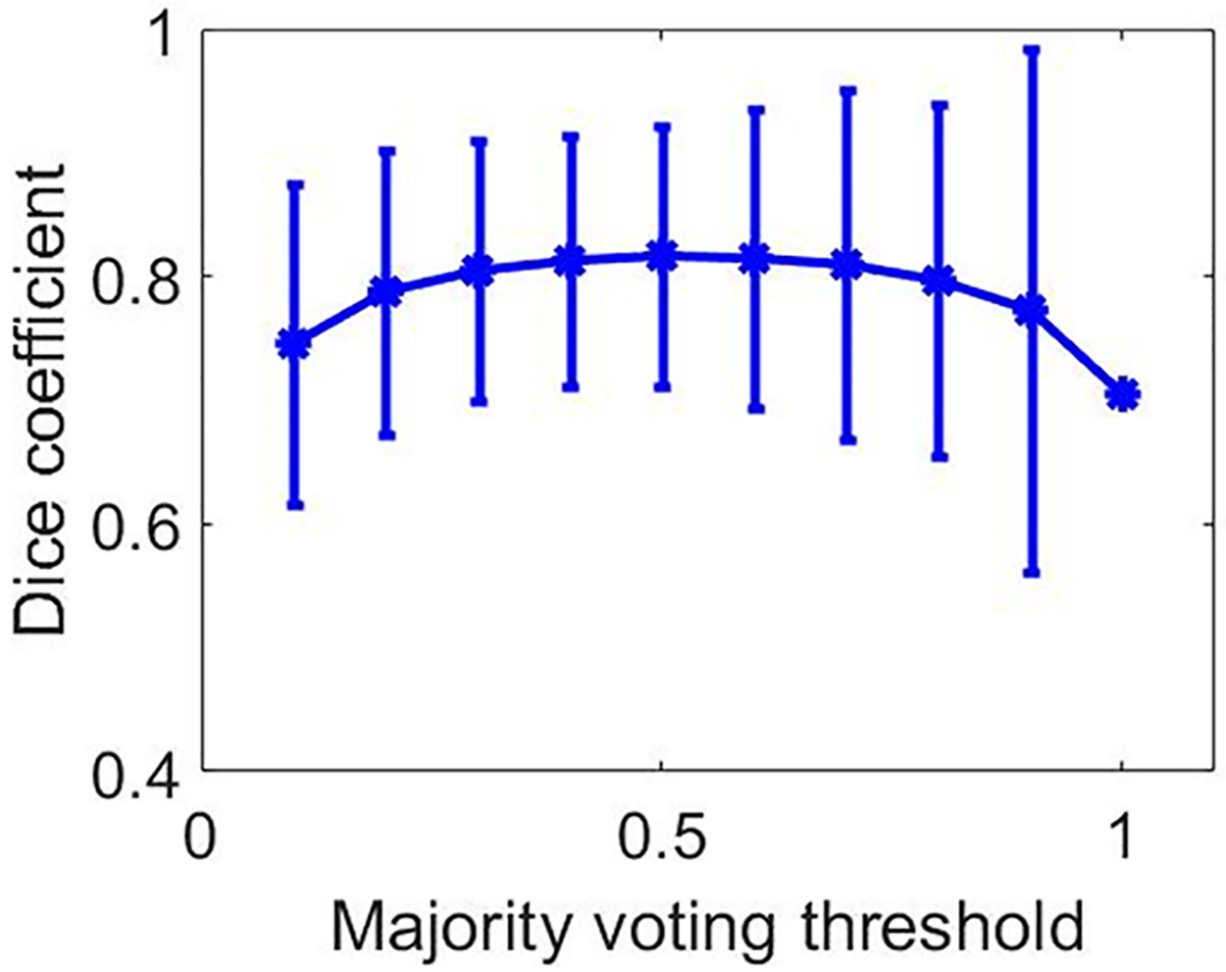
Dice coefficient values for different majority voting thresholds along with error bars.

The mean and standard deviation value of the Dice coefficient, TPF, and FPF achieved during testing is presented in Table 5. Fig 2 shows the performance of the three parameters for Multi U-net and DRLS algorithm against the benign and malignant pathologies along with fibroadenoma and invasive ductal carcinoma, which are the two predominant pathologies in their class. More detailed graphs visualizing all pathologies with more than 10 samples for multi U-net and DRLS algorithm are available in S2 Fig. The same sets of parameters are visualized against different BI-RADS for both Multi U-net and DRLS in Fig 3. Fig 4 depicts the variation in mean value of the Dice coefficient against the majority voting threshold value. A two-tailed P value of P < 0.0001 was obtained for the comparison between multi U-net and original U-net algorithm, showing significant improvement over the original U-net algorithm. To showcase the strengths and weakness of the algorithm, six review cases are presented.

**Table 5:** Mean and standard deviation values for different metrics of the test cases of Multi U-net algorithm (MU). Same metrics for DRLS algorithm (DRLS) and original U-net (OU) are also shown.

Metrics		All cases (n = 61)	Benign (n = 39)	Malignant (n = 22)	IDC (n = 14)	Fibroadenoma (n = 23)
Dice coefficient	MU	0.82±0.10	0.81±0.11	0.83±0.09	0.81±0.10	0.84±0.09
	DRLS	0.84±0.09	0.82±0.10	0.87±0.07	0.87±0.06	0.84±0.06
	OU	0.52±0.27	0.48±0.28	0.57±0.24	0.55±0.28	0.48±0.27
TPF^a^	MU	0.84±0.15	0.80±0.16	0.89±0.11	0.90±0.13	0.80±0.14
	DRLS	0.79±0.12	0.76±0.12	0.83±0.12	0.83±0.12	0.77±0.10
	OU	0.61±0.06	0.55±0.06	0.70±0.05	0.68±0.07	0.57±0.04
FPF^b^	MU	0.01±0.02	0.01±0.02	0.02±0.02	0.02±0.02	0.01±0.01
	DRLS	0.01±0.02)	0.01±0.02	0.01±0.02	0.01±0.02	0.01±0.01
	OU	0.31±0.06	0.31±0.05	0.27±0.07	0.29±0.09	0.32±0.04

^a^True positive fraction

^b^False positive fraction

### Review of selected cases

The results of 6 different cases are reviewed to demonstrate the ability and the limitations of the algorithm.

**Fig 5:**
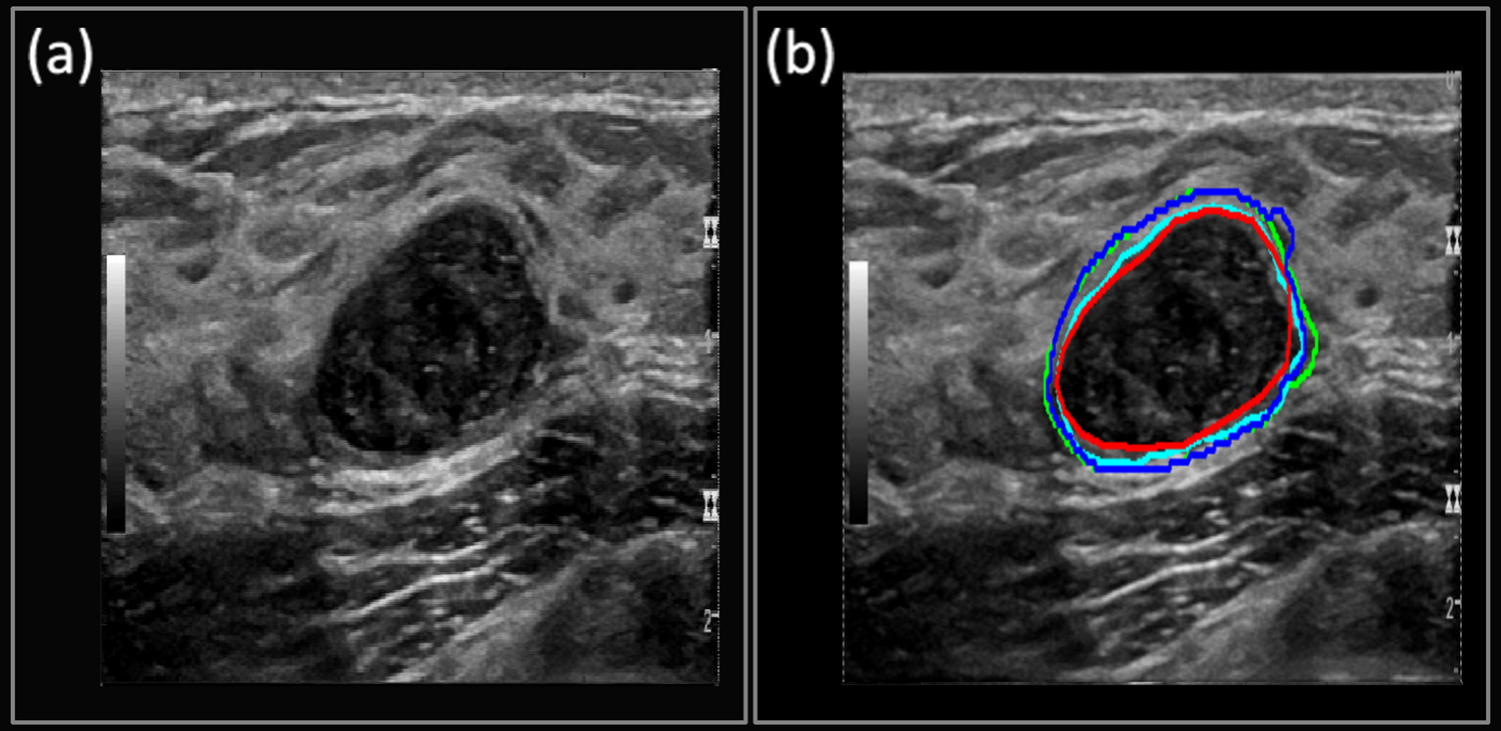
(a) B-mode Image of **b**enign-cellular fibroepithelial Mass. (b) The manually segmented boundary is shown in red, the Multi U-net predicted boundary is shown in blue, the DRLS predicted boundary is shown in green and original U-net is shown in cyan.

**Case 1**: The suspicious mass from Fig 5(a) shows the B-mode image of a benign-cellular fibroepithelial mass. The mass has typical smooth boundaries of a benign mass and is oval in shape. Fig 5(b) shows the manually segmented boundary in red, Multi U-net predicted boundary in blue and DRLS predicted boundary in green. Dice coefficient for Multi U-net algorithm was 0.94 and for DRLS algorithm was 0.90. Performance of original U-net algorithm is at par with Multi U-net algorithm with a Dice coefficient of 0.94. Typical benign cases are easily segmented by the Multi U-net algorithm; however, the Multi U-net algorithm overestimates the boundary size in comparison to DRLS algorithm. DRLS algorithm does not select the hyperechoic region however; it also underestimates the hypoechoic region.

**Fig 6:**
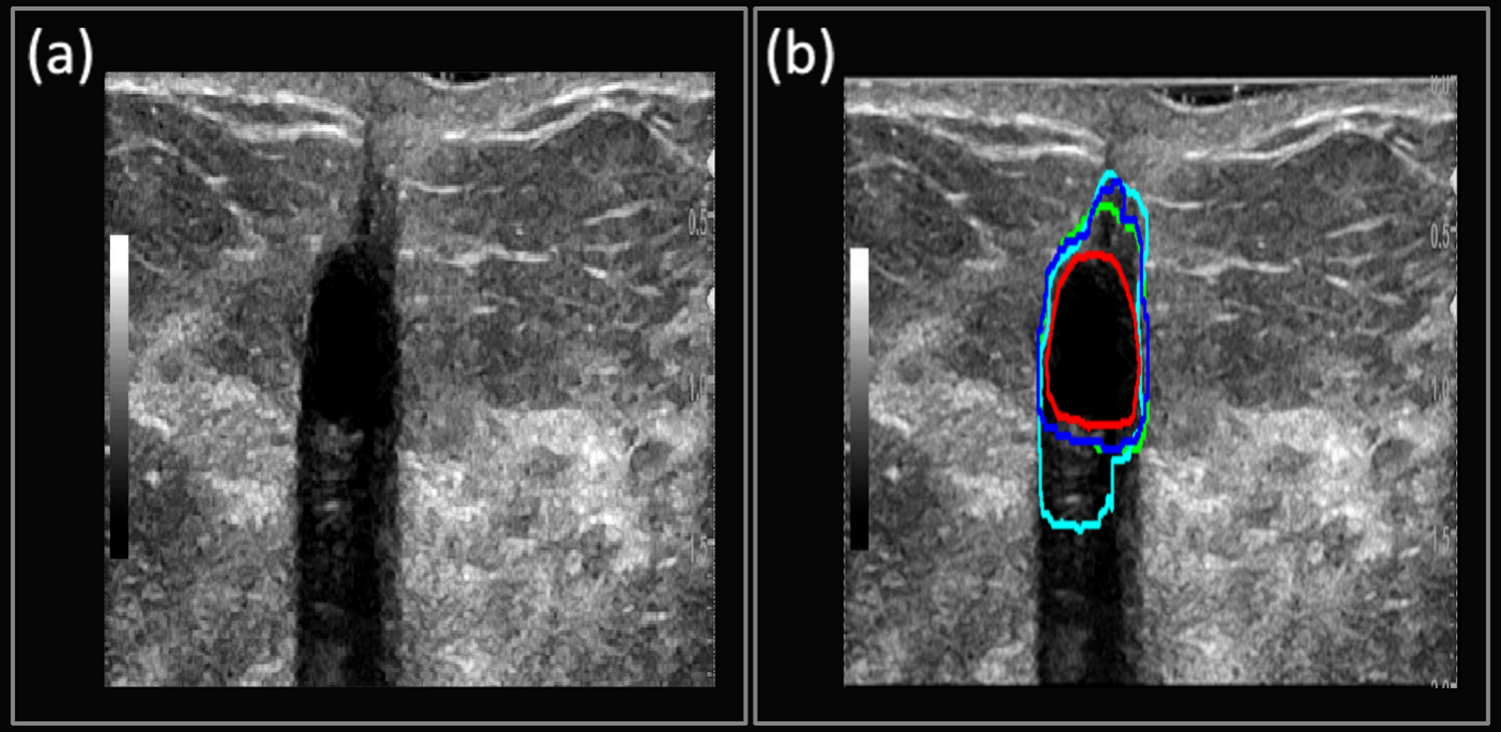
(a) B-mode Image of benign fat necrosis. (b) Manually segmented boundary is shown in red, Multi U-net predicted boundary is shown in blue, the DRLS predicted boundary is shown in green and original U-net is shown in cyan.

**Case 2**: The suspicious mass from Fig 6(a) depicts the B-mode image for benign fat necrosis with dystrophic calcifications. Notice the posterior acoustic shadowing beneath the benign mass, which is an unusual feature for benign masses. Fig 6(b) shows the ability of the Multi U-net algorithm to differentiate the posterior shadowing from the suspicious mass (Dice coefficient of 0.88), although the size of the boundary is overestimated. The DRLS algorithm (Dice coefficient of 0.88) overestimates by including some of the posterior acoustic shadowing region while doing a better job in estimating the anterior part of the suspicious mass. Performance of original U-net algorithm is poorer than Multi U-net algorithm with a Dice coefficient of 0.64.

**Fig 7:**
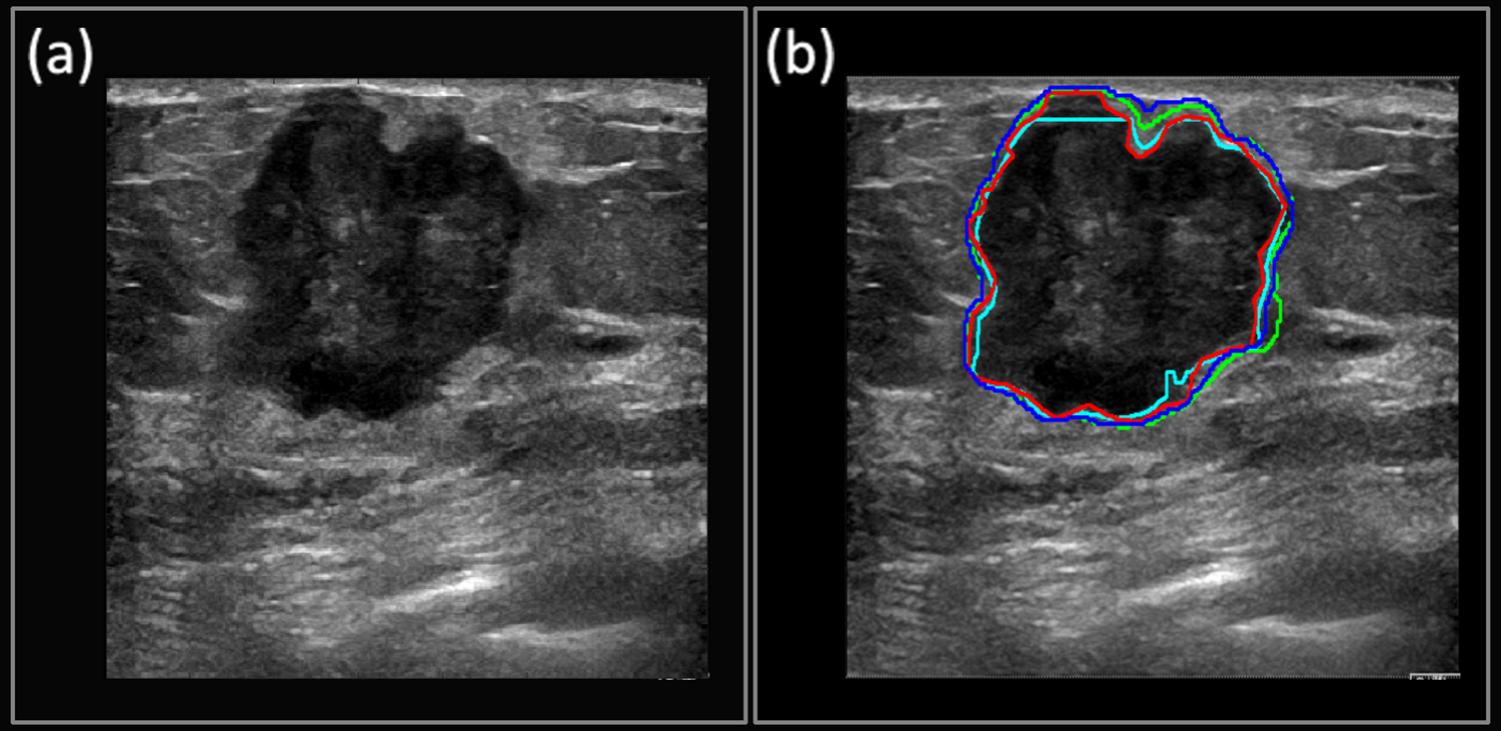
(a) B-mode Image of invasive/infiltrating ductal carcinoma. (b) Manually segmented boundary is shown in red, Multi U-net predicted boundary is shown in blue, the DRLS predicted boundary is shown in green and original U-net is shown in cyan.

**Case 3**: The suspicious mass from Fig 7(a) was pathologically confirmed as a malignant invasive/infiltrating ductal carcinoma, grade III. The irregular boundaries of the mass are typical of malignant masses and are usually challenging cases for segmentation algorithms. Fig 7(b) shows how the Multi U-net algorithm is capable of capturing majority of the intricate details belonging to the irregular boundaries (Dice coefficient of 0.88). The performance of DRLS (Dice coefficient of 0.95) is comparable to Multi U-net however; the posterior and left side boundaries are smoother when compared to Multi U-net. Performance of original U-net algorithm is slightly better than Multi U-net algorithm with a Dice coefficient of 0.96.

**Fig 8:**
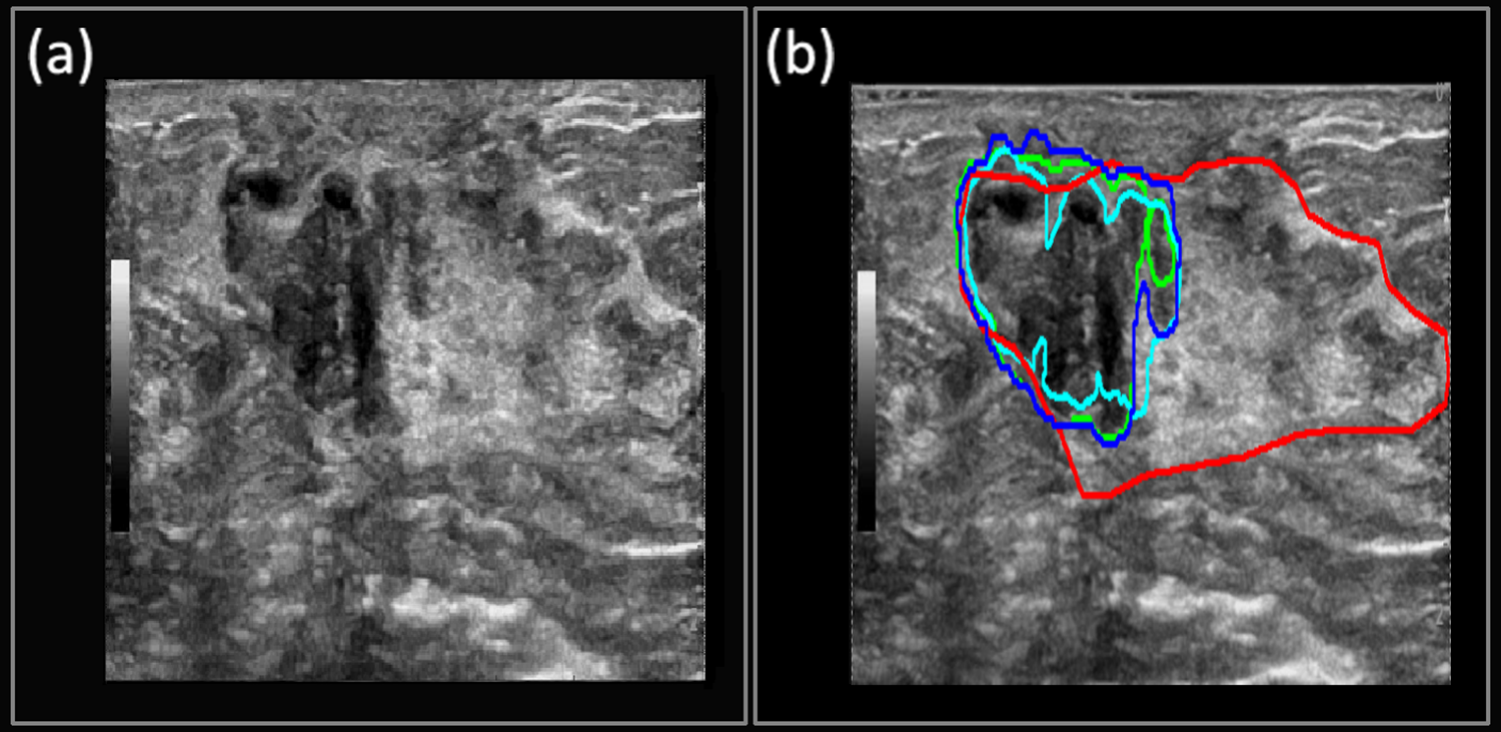
(a) B-mode Image of **f**ibroadenoma with mild usual ductal hyperplasia and apocrine cysts. (b) Manually segmented boundary is shown in red, Multi U-net predicted boundary is shown in blue, the DRLS predicted boundary is shown in green and original U-net is shown in cyan.

**Case 4**: The suspicious mass from Fig 8(a) was confirmed as a fibroadenoma with mild usual ductal hyperplasia and apocrine cysts. The suspicious mass covers the majority of the field of view and has a hypoechoic part on the left side of the image, and a hyperechoic part on the right-hand side. As seen in Fig 8(b), the Multi U-net algorithm identifies the apocrine cysts but fails to identify the mild usual ductal hyperplasia which can be seen as the hyperechoic mass on the right, resulting in a Dice coefficient of 0.44. DRLS algorithm has an edge over the Multi U-net algorithm (Dice coefficient of 0.85) due to the initial seed, which is just an eroded version of the correct mask, and is able to identify majority of the mild usual ductal hyperplasia. Performance of original U-net algorithm is slightly better than Multi U-net algorithm with a Dice coefficient of 0.49.

**Fig 9:**
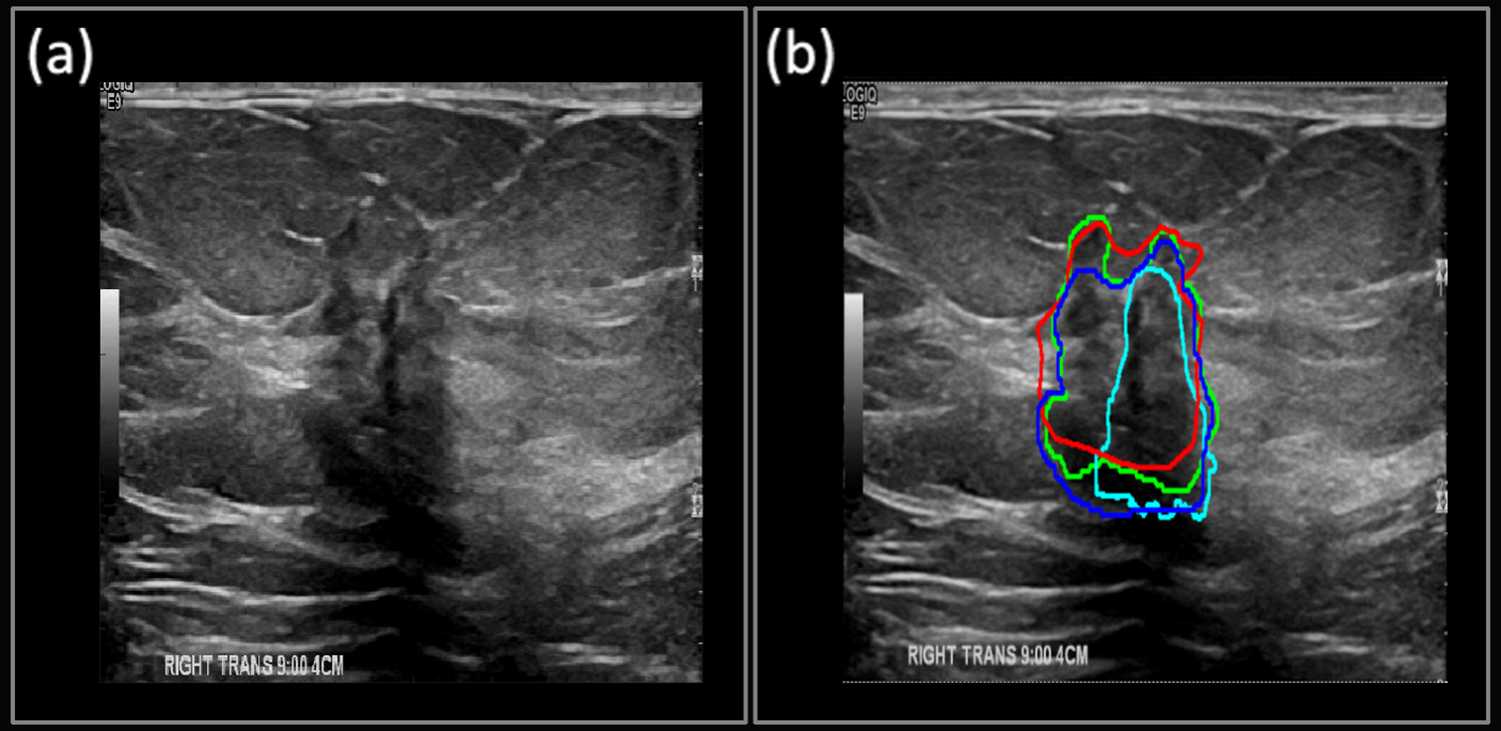
(a) B-mode Image of invasive mammary carcinoma. (b) Manually segmented boundary is shown in red, Multi U-net predicted boundary is shown in blue, the DRLS predicted boundary is shown in green and original U-net is shown in cyan.

**Case 5**: The suspicious mass from Fig 9(a) was confirmed as invasive mammary carcinoma with mixed ductal and lobular features, grade I-II. Perineural invasion is also identified. Fig 9(b) depicts how the Multi U-net algorithm predicts the hypoechoic region as part of the suspicious mass but fails to select the hyperechoic halo (Dice coefficient of 0.64). The performance of DRLS algorithm (Dice coefficient of 0.87) is better than Multi U-net algorithm along with partial identification of the hyperechoic halo. Performance of original U-net algorithm is slightly lower than Multi U-net algorithm with a Dice coefficient of 0.52.

**Fig 10:**
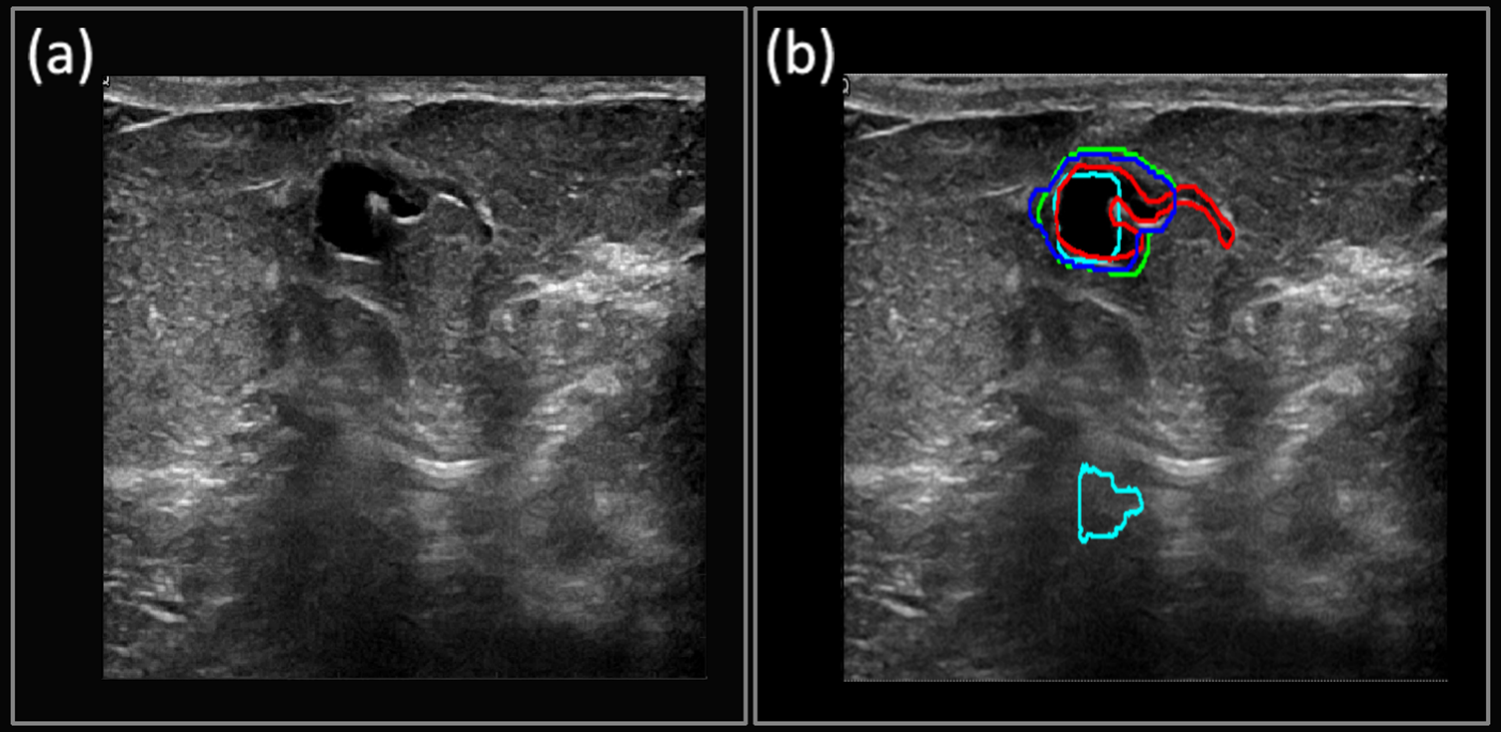
(a) B-mode Image of **c**lustered apocrine cysts. (b) The manually segmented boundary is shown in red, Multi U-net predicted boundary is shown in blue, the DRLS predicted boundary is shown in green and original U-net is shown in cyan.

**Case 6**: The suspicious mass from Fig 10(a) was pathologically confirmed as clustered apocrine cysts, with proliferative fibrocystic changes including usual ductal hyperplasia. Fig 10(b) shows that the Multi U-net algorithm fails to segment the sharp extension of the cyst but detects the central region of the cyst, along with the hypoechoic region surrounding it (Dice coefficient of 0.78). DRLS algorithm (Dice coefficient of 0.74) also fails to identify the sharp extension of the cyst and performs poorer than Multi U-net due to the initial seed, which did not include the cystic extension. Performance of original U-net algorithm is lower than Multi U-net algorithm with a Dice coefficient of 0.56. The original U-net algorithm identifies an isolated region beneath the suspicious mass. However, the Multi U-net algorithm is able to avoid that as this outlying region may exist in only a few U-nets and the majority voting can remove such outlying regions.

## Discussion

The paper presents the performance of Multi U-net segmentation algorithm for suspicious breast masses. The Multi U-net algorithm segments the test images in real time with a mean Dice coefficient of 0.82, which is on par with other seed-based segmentation algorithms (DRLS, Dice coefficient of 0.84). The performance of original U-net algorithm is significantly poorer than Multi U-net algorithm even though the image size used in training of original U-net algorithm is larger than Multi U-net algorithm. Unlike its contemporary seeded algorithms, the Multi U-net algorithm does not require an initial seed and could be used in applications requiring minimum user interaction. The abilities and the limitations of the algorithm are exhibited through the help of selected review cases. The complexity in a deep learning algorithm does not depend on the algorithm itself, but in the data that is used to train the algorithm. Deep learning algorithm’s performance increases as the algorithm is trained on more diverse and unique cases. The data gathered from the past and futures studies can be further used to improve the performance of the algorithm.

As shown in Table 1, the majority of the data used for training the algorithm falls under BI-RADS 4 category. BI-RADS 4 consists of suspicious cases with a high variability in malignancy rate (3%-94%) [31]. Cases that fall under the category of BI-RADS 4 are challenging for segmentation compared to BI-RADS 2, 3, and 5, which are more well-defined cases. The algorithm was trained mostly on the features of BI-RADS 4 cases, which may limit its ability to learn the typical characteristics of benign or malignant masses. This was one of the major limitations for training the algorithm. A wider variety of BI-RADS cases would enable the algorithm to learn characteristics of typical benign and malignant pathologies, thus enabling better performance.

From Fig 2(a) and 2(b), we observed that the median Dice coefficient and TPF are higher for malignant pathologies compared to benign pathologies; however, FPF was higher for malignant pathologies compared to benign pathologies (Fig 2(c)), which implies that the algorithm overestimated the suspicious boundary for malignant masses, whereas the algorithm underestimated the benign masses. Benign masses are usually easier to segment than malignant masses however, the lower Dice value for benign masses stems from the fact that the training data had very few typical benign cases as the patient pool consists of patients undergoing biopsy. The performance of two dominant pathologies (i.e., fibroadenoma and invasive ductal carcinoma) closely follows their respective biopsy classes. When comparing the Multi U-net algorithm performance to DRLS algorithm the median value of Dice coefficient is comparable for benign cases and not malignant cases however; the spread in Dice values for DRLS algorithm is always smaller than Multi U-net algorithm. The TPF and FPF for DRLS algorithm is always lower than Multi U-net, implying that DRLS is always underestimating the suspicious mass whereas Multi U-net is overestimating.

From Fig 3(a) and 3(b), it is evident that the performance of the Multi U-net algorithm was better for BI-RADS 3 and 4 compared to BI-RADS 5. Suspicious masses with irregular shapes and margins are usually assigned BI-RADS 5, and are difficult cases for the algorithm to capture the intricate details. Similar to our observation from Fig 2 we observe that the TPF for DRLS algorithm is higher or comparable to Multi U-net algorithm. However, the FPF for DRLS is lower than Multi U-net algorithm which implies the overestimation of suspicious mass by Multi U-net and under estimation by DRLS.

The performance of DRLS algorithm is highly dependent on the initial seed as shown in case 4 and case 6. The DRLS algorithm has leverage over the Multi U-net algorithm in our implementation because the initial seed was just an eroded version of the manually segmented expert’s mask. This was intentionally done to highlight the performance of Multi U-net algorithm when the seed has been selected by a user with good knowledge about the suspicious mass. However, the initial seed may not work in favor of DRLS algorithm when the size of the suspicious mass is too small or if the mass has spiculations, narrow extensions or irregular boundaries.

The better performance of original U-net algorithm for case 3 and case 4 can be attributed to the larger image size used in training the algorithm compared to Multi U-net which has a coarser resolution, as the contours are scaled to the original image size before overlaying on the B-mode image. For majority of the review cases the performance of original U-net is poorer than Multi U-net algorithm. This improvement in Dice coefficient can be mainly attributed to ten-fold cross validation and majority voting technique.

Selection of majority voting threshold is important for optimizing the performance of ten cross fold validated Multi U-net algorithm. Since, the initialization for all the ten U-nets is random they converge to different local minima’s, resulting in ten unique maps for the same image. At lower values of majority voting threshold the algorithm is overestimating the suspicious mass region resulting in low Dice coefficient values as seen in Fig 4. At higher values of majority voting threshold the algorithm is underestimating the suspicious mass again resulting in low Dice coefficient values as seen in Fig 4. A balance between overestimating and underestimating the suspicious mass is observed at 0.5 majority voting threshold.

**Fig 11:**
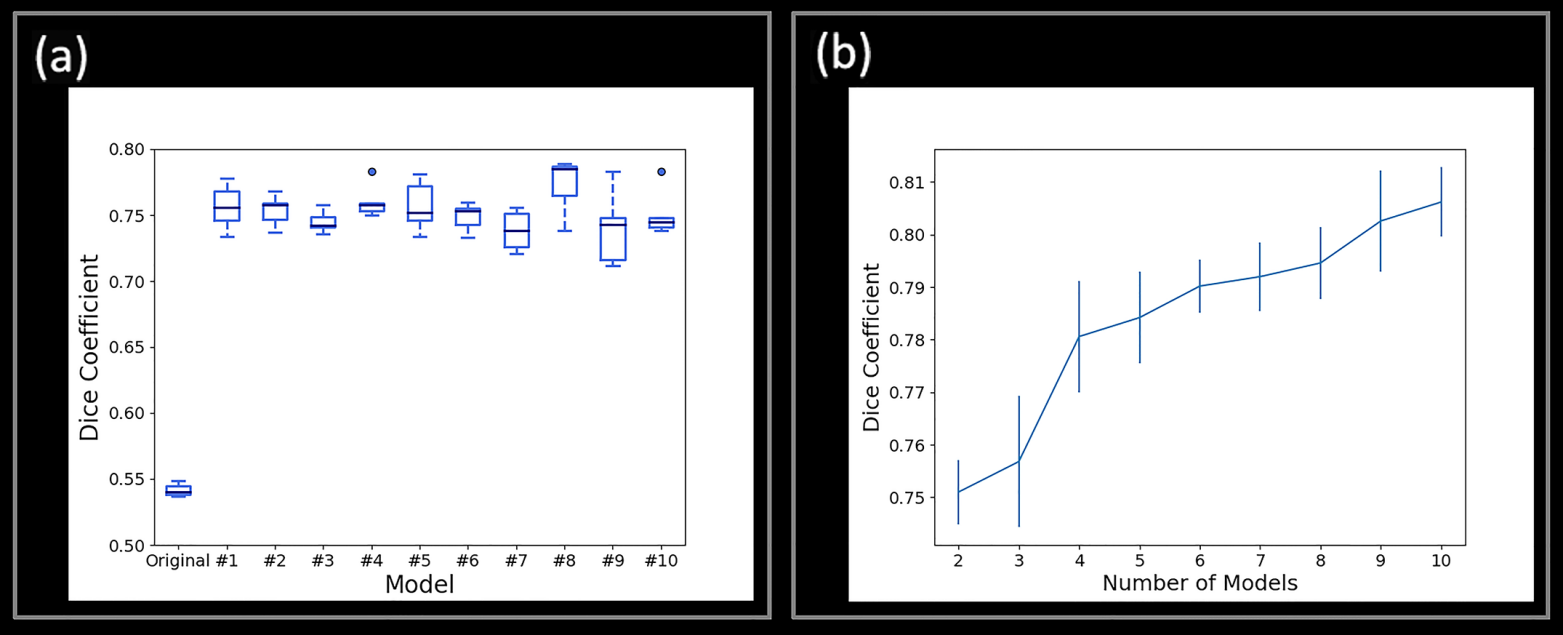
(a) Bar plot comparing Dice coefficient between original U-net and ten folds of Multi U-net. Each fold is evaluated 5 times to show the variance within each fold. (b) Error bars showing the increasing performance of U-net as more models are included in majority voting. Five different U-nets models are evaluated to show the variance.

The advantage of using ten-fold cross-validated Multi U-net models over original U-net can be seen in Fig 11(a). The Dice coefficient for original U-net is 0.54 which is low compared to the ten folds of Multi U-net. Each fold of the Multi U-net has been repeated 5 times to show the error bar in Fig 11(a). The variance of each fold is very small as seen in Fig 11(a). An increase of nearly 0.20 Dice coefficient can be observed for each individual fold of Multi U-net when compared to the original u-net. The performance can be further increased by using multiple cross folds followed by majority voting as shown in Fig 11(b). The Dice coefficient increases from 0.75 to 0.81 as the number of models used for majority voting increases from two to ten. A majority threshold criterion of 0.5 is used for all the folds.

The original U-net is prone to predicting a larger region than the actual lesion. The penultimate output of the U-net algorithm assigns probability (probability that the pixel belongs to the lesion) to each pixel which is then converted into a binary probability using the sigmoid classifier. The center of the segmented part is predicted with higher probability. However, the periphery pixels have lower probability thus lower confidence and are prone to be spurious. Using multi U-net enables to increase the probability of the pixels on the periphery by trimming the periphery based on a majority voting threshold. The performance of original U-net is poor compared to multi U-net algorithm. However, the performance of original U-net algorithm with the same hyper parameters as multi U-net algorithm improves as seen in model 1 to 10 from Fig 11(a). The dice coefficient of original U-net with same hyper parameter is 0.76±0.02. The incremental improvement from original U-net with same hyper parameters as multi U-net algorithm can be seen in Fig 11(b).

Hypoechoic suspicious masses are sometimes surrounded with hyperechoic boundaries. There is an ongoing deliberation on the selection of these hyperechogenic regions as part of a suspicious mass [32], as seen in Fig 9(a). The decision to include the hyperechoic region as a part of the suspicious mass is taken after reviewing the suspicious mass from different angles and orientations. Acquiring more images of the same mass can enable the algorithm to better segment the suspicious mass. Fig 8(a) is a similar case, with a hyperechoic region on the right-hand side of the image. The hyperechoic mass is a mild usual ductal hyperplasia. The poor performance of Multi U-net algorithm can be explained due to the limited training size of clustered apocrine cysts (maximum of 7 cases, the word maximum is used as it is unknown how may samples are in training and validation set for each crossfold) and hyperplasia (maximum of 2 cases). Apocrine cysts usually have more contrast and have features which are shared with other pathologies. However, the hyperplasia has lower contrast and may have textural features which are not shared with other pathologies. Adding more cases of ductal hyperplasia will help in improving the performance of Multi U-net algorithm. The Multi U-net algorithm fails to properly segment the cystic mass, shown in Fig 10. Cysts are usually characterized with a hypoechoic mass in the middle. The cyst presented in Fig 10 is unique as it has an irregular boundary. The algorithm latches onto the hyperechoic region on the top right side of the cyst and estimates that region to be the boundary of the cyst.

The training time for the algorithm was 172 seconds per epoch on Tesla K40C. Table 6 shows the inference time per image in milliseconds. The small processing time for inferencing location of the suspicious mass allows the algorithm to be used in live imaging. Modern US machines with plane wave imaging capability use GPUs for beamforming as the data size is large. The preinstalled GPUs on US machines can be leveraged to segment the suspicious mass in live imaging. The testing time can be further reduced to provide live imaging by reducing the number of filters. Using GPUs with higher single precision Tflops can further increase the number of images inferenced in a second. Titan xp which has higher single precision Tflops than Tesla k40c performs nearly four times faster as shown in Table 6.

**Table 6:** Inference time in milliseconds for different GPU’s.

GPU	Inference time per image in ms
Tesla k40c	55.29
Titan xp	13.83
GTX 960	831.64

Advancements in suspicious mass segmentation offer potential benefits in their classification. Additionally, deep learning techniques that seek to diagnose suspicious masses are heavily dependent on accurate segmentation to capture boundary-related features. Improved accuracy in real-time automatic segmentation will enable the development of live automatic classification of suspicious masses. Unlike classification algorithms, which need bigger data sizes, segmentation algorithm can work with smaller data size as the sample size of the data is not just the number of images but the number of images multiplied by image size as each pixel is individually classified into normal or suspicious mass.

## Limitations

Clinical 2D US images provide only a planar view of the *in-vivo* tissue. The planar view of the tissue varies with angle, orientation and pre-compression. The same mass appears different, depending on how the above mentioned parameters are changed. The manual segmentation masks were created by a sonographer who had access to live imaging and thus viewed the suspicious mass by sweeping over it in real time as well as rocking, heeling, and toeing to view it from different angles, orientations, and at different pre-compression levels. Pre-compression changes the contrast of the mass with respect to the surrounding tissue. The angle of inclination changes the posterior acoustic shadowing and enhancement. Orientation changes the cross-section that is being examined. The Multi U-net algorithm has access to only a single frame, which limits the ability of the algorithm to better delineate the boundaries of the suspicious mass. Also, currently the algorithm treats the images from different cross-sections of the same suspicious mass as independent cases; thus, the information from different cross-sections is not combined. More images of the same suspicious mass acquired with different imaging parameters can improve the performance of the algorithm further. Unique pathologies which can be mixture of two different pathologies are not readily available in the small training set and are hard to segment as shown in case 4. A larger training set inclusive of various pathologies can further improve the performance of segmentation algorithms.

## Conclusion

The Multi U-net algorithm can segment suspicious breast masses in real time without the need for an initial seed, and performs on par with contemporary seeded algorithms (DRLS). A significant improvement is obtained over original U-net by using the multi U-net algorithm. The increment is due to combined contribution of better hyper parameter selection and use of ten-fold cross validation technique. The performance of the algorithm can be further improved with a bigger dataset and can be extended to diagnosis of suspicious masses in the future. The algorithm is independent of US machine and can be used in any commercially available clinical system.

## Supporting information

S1 Fig Schematic diagram showing the ten-fold cross validation technique for splitting data into training, validation and testing set. The testing set is never used for training and validation. The training and validation set are split into ten different parts with validation set being different for each of the ten U-net models.
(TIF)

S2 Fig Boxplot showing the performance of Multi U-net and DRLS algorithm for (a) Dice Coefficient, (b) TPF, and (c) FPF for benign, fibroadenoma, fibrocystic changes, papilloma. (d) Dice Coefficient, (e) TPF, and (f) FPF for Malignant, Invasive Ductal Carcinoma, Invasive Lobular Carcinoma, Invasive Mammary Carcinoma. TPF indicates true positive fraction; FPF indicates false positive fraction.
(TIF)
