# Supplementary figures and images for: Automated and real-time segmentation of suspicious breast masses using convolutional neural network
Source: PLoS One. 2018 May 16;13(5):e0195816. doi: 10.1371/journal.pone.0195816 (PMC5955504; doi:10.1371/journal.pone.0195816)

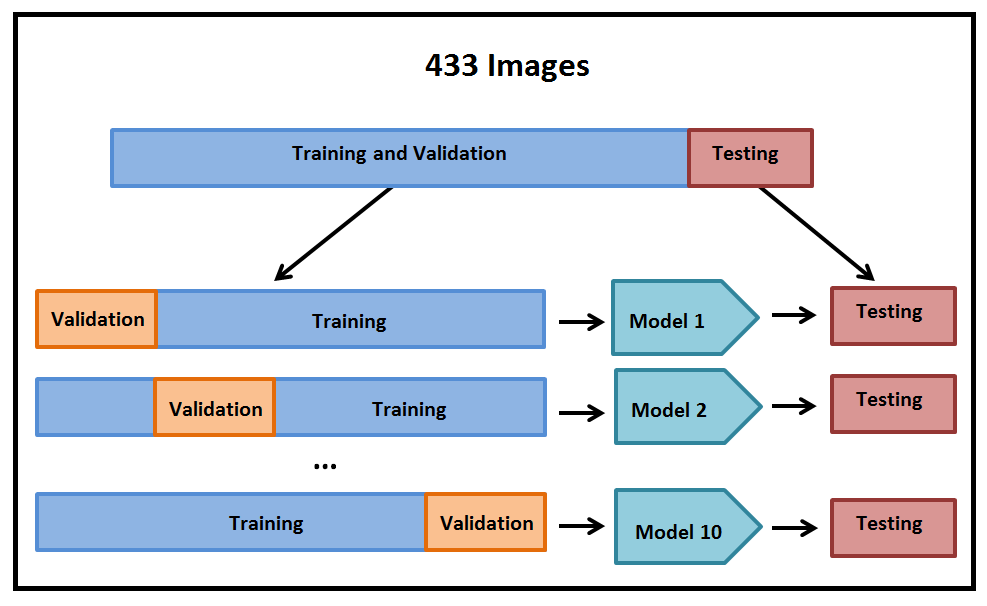

Supplement: S1 Fig — The testing set is never used for training and validation. The training and validation set are split into ten different parts with validation set being different for each of the ten U-net models. (TIF) [file pone.0195816.s001.tif]

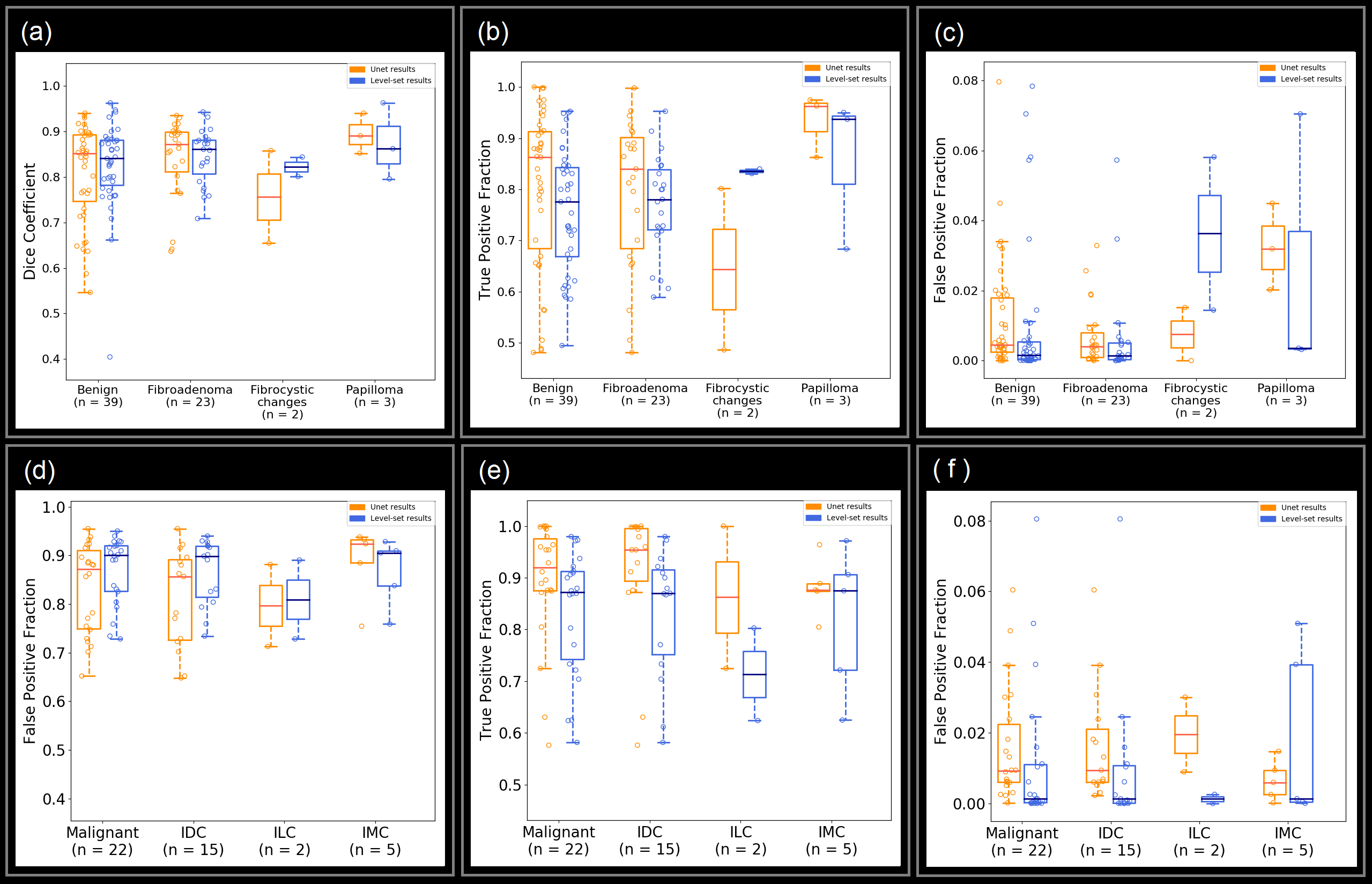

Supplement: S2 Fig — Boxplot showing the performance of Multi U-net and DRLS algorithm for (a) Dice Coefficient, (b) TPF, and (c) FPF for benign, fibroadenoma, fibrocystic changes, papilloma. (d) Dice Coefficient, (e) TPF, and (f) FPF for Malignant, Invasive Ductal Carcinoma, Invasive Lobular Carcinoma, Invasive Mammary Carcinoma. TPF indicates true positive fraction; FPF indicates false positive fraction. (TIF) [file pone.0195816.s002.tif]
